# Clinical and functional outcomes of upper extremity 3D-printed immobilization devices compared to traditional methods: a systematic review with limited meta-analysis

**DOI:** 10.1186/s13018-026-07109-3

**Published:** 2026-07-10

**Authors:** Alexis Watson, Samuel Hauke, Kyle DeRoma, Royal Shrestha, Anuj Vimawala, Michael Lohse, Asiya Falak, Wen Chao, Al Smith, Thomas Murphy, Rand Kittani

**Affiliations:** 1 Carle Illinois College of Medicine, University of Illinois Urbana-Champaign, Urbana, IL USA; 2 Carle Health, Carle McLean County Orthopedics, Urbana, IL USA; 3 Penn Medicine, University of Pennsylvania Health System, Philadelphia, PA USA

**Keywords:** 3D printing, Cast immobilization, Fracture healing, Orthoses, Patient satisfaction, Functional recovery

## Abstract

**Background:**

Fractures are a common cause of emergency department visits, and immobilization choice affects not only fracture healing but also comfort, hygiene, mobility, and quality of life. Conventional casting remains standard but is associated with limitations, including bulk, discomfort, and poor water tolerance. 3-Dimensional (3D)-printed, patient-specific immobilization may improve ventilation and functional tolerance while maintaining stability, but clinical evidence is still emerging and heterogeneous. This systematic review aims to evaluate the current clinical evidence on 3D-printed immobilization technologies versus traditional standard methods for fracture management in both pediatric and adult patients with respect to fracture healing, stability, pain, and functional recovery.

**Methods:**

A comprehensive search of PubMed, Scopus, and Web of Science databases was performed from database inception until November 2025 on the topic of evaluating 3D-printed technology to create casts, splints, or orthoses for upper-extremity fracture immobilization. Due to heterogeneity in design, interventions, and outcome reporting, results were synthesized narratively.

**Results:**

Of 828 articles retrieved from the initial search, 581 studies were included for screening after removing duplicate articles, and underwent title and abstract screening, with 32 full-text studies assessed for eligibility. Thirteen studies met full inclusion criteria, while 19 studies were excluded. Twelve (n = 456) were included in the result analysis as they all focused on upper-extremity fractures, all of which had a moderate-to-high risk of bias. Fracture union was reported at or near 100% across studies. Patient satisfaction generally favored 3D-printed immobilization (10/11 studies), with a non-significant large, pooled effect (Hedges’ g = 0.83; 95% CI = 0.21–1.88; *p* = 0.085; I^2^ = 77.5%). Pain showed a non-significant small-to-moderate benefit (g = 0.36; 95% CI = 1.94–2.66), and functional outcomes were similar between groups (g = 0.13; 95% CI = 1.02–1.28). Skin-related outcomes more often favored 3D-printed devices (7/9 studies), with reduced irritation and hygiene burden, while device weight was consistently lower, but cost and production time were higher.

**Conclusion:**

3D-printed immobilization devices show comparable fracture healing and improved patient satisfaction and skin tolerability compared with conventional casting, with early functional advantages that are not consistently sustained long term. However, the evidence is limited by small sample sizes, short follow-up, moderate-to-high risk of bias, and substantial heterogeneity, resulting in low-to-moderate certainty. Larger, high-quality randomized trials with longer follow-up are needed to confirm these findings and inform clinical recommendations.

**Trial registration:**

Protocol registered prospectively in an international systematic review registry (PROSPERO) (registration number: CRD420251182244).

## Background

Upper extremity fractures are extremely common in the emergency department (ED) with an annual incidence of approximately 67.6 per 10,000 persons in the United States, which, in a population of 87 million Americans, is roughly 590,000 upper extremity fractures annually [1]. Distal forearm fractures alone account for approximately 17% of all ED presenting fractures [2]. Despite sustaining serious injuries, patients are often most concerned with recovery time, hygiene, mobility, and the impact on overall quality of life, especially in the athletic and active population [3]. These factors are heavily influenced by the type of immobilization device used. Currently available casting options include plaster, synthetic materials, and, more recently, 3D-printed designs.

Plaster casts remain widely used for fracture immobilization due to their excellent moldability, ease of application, and modifiability. First described in 1852 by a Dutch military surgeon, the fundamental principles of plaster‑based immobilization remain largely unchanged despite material evolution [4]. However, plaster casts present distinct disadvantages, including bulkiness, heavy weight, low durability, poor water resistance, and a higher risk of cast‑related skin complications [4]. Synthetic casts were subsequently introduced as an alternative, offering lighter weight, greater durability, faster setting times, and reduced bulk. Yet, despite these advantages, synthetic options are less moldable and more difficult to modify once set.

Given the limitations of both plaster and synthetic options, 3D-printed immobilization devices have emerged as a novel alternative utilizing a wide range of advanced materials. Utilized clinically for approximately 15 years, 3D-printed devices offer unique advantages centered around patient-specific fit and customization [5]. These devices are lighter, highly ventilated, and have demonstrated noninferior clinical efficacy and recovery timelines when compared with standard‑of‑care immobilization [6].

Recent randomized trials report early improvements in function and pain relief with customized 3D-printed casts compared with traditional casts [7, 8]. Prior systematic reviews similarly suggest several potential advantages of 3D-printed orthoses, including improved fit, comfort, ventilation, and patient satisfaction, while demonstrating biomechanical performance (e.g., stiffness) that is generally comparable to conventional plaster casts [9]. In addition, skin-related outcomes appear favorable in most reported studies. Collectively, the available evidence suggests that 3D-printed immobilization devices may offer clinically meaningful advantages in patient comfort and usability, while maintaining adequate mechanical performance relative to standard immobilization [10, 11].

However, the existing evidence base remains limited and heterogeneous. Most studies are small, single-center trials that vary considerably in study design, patient populations, fracture types, device designs, and outcome measures, thereby limiting overall comparability and generalizability. Consequently, uncertainty remains regarding the consistency of these benefits across different clinical settings, indications, and age groups. Despite promising findings reported in academic and specialist centers, widespread clinical adoption remains constrained, and real-world implementation has not been well characterized [12, 13].

Additional barriers to widespread implementation include high 3D-printing infrastructure costs, a lack of standardized software and scanning tools, longer production times, and the need for specialized staff training [14, 15, 16]. The clinical utility of these devices is further obscured by a lack of adequately powered multicenter studies, alongside inconsistent study parameters, such as varying follow-up periods, assessment tools, material compositions, and cost analyses, which limit the ability to synthesize findings meaningfully across the literature [7, 17–20].

Given these gaps, there remains a critical need to systematically synthesize the current evidence to better define the clinical effectiveness, safety, and real-world applicability of 3D-printed immobilization devices. Therefore, this study aims to systematically review the current clinical evidence on 3D-printed immobilization technologies versus traditional standard methods for fracture management across both pediatric and adult patients, accounting for various device types (casts, splints, and orthoses).

## Methods

### Study design

A systematic review was conducted in accordance with Preferred Reporting Items for Systematic Reviews and Meta-Analyses (PRISMA) guidelines. The protocol was registered prospectively within PROSPERO (registration number: CRD420251182244).

Comprehensive searches were conducted in PubMed, Scopus, and Web of Science from inception to November 2025. The search targeted studies in English, either native or translated. The Boolean search strings were tailored to the syntax requirements of each database (Appendix A). In Web of Science, a topic-field search (TS) was employed, combining terms related to 3D printing technology (“3D printed” OR “3D-printed” OR “three-dimensional printed” OR “three dimensional printed”), device type (cast* OR splint* OR orthos*), and anatomical region or clinical context (wrist OR hand OR finger OR distal radius OR fracture* OR carpometacarpal OR postoperative), yielding 451 results. The same conceptual framework was applied in Scopus using the TITLE-ABS-KEY field operator, which retrieved 93 records. In PubMed, an equivalent search was conducted using free-text terms across all fields, returning 284 records. The combined search yielded a total of 828 records before deduplication. Truncation symbols (e.g., cast*, splint*, orthos*, fracture*) were used to capture variant word forms, and all three concept blocks related to the AND operator to ensure that retrieved studies addressed 3D printing, the relevant device category, and the upper-extremity clinical context simultaneously.

### Inclusion and exclusion criteria

Eligible studies included studies evaluating 3D-printed casts, splints, or orthoses used for fracture management. Additional inclusion criteria included: human clinical studies (randomized controlled trials, non-randomized controlled trials, prospective or retrospective cohorts, case–control studies, and case series with N ≥ 5), studies where the intervention is a 3D-printed cast/splint used for patient fracture immobilization, studies reporting ≥ 1 pre-specified primary or secondary outcome, and studies available in the English language, both native or translated.

**Fig. 1:**
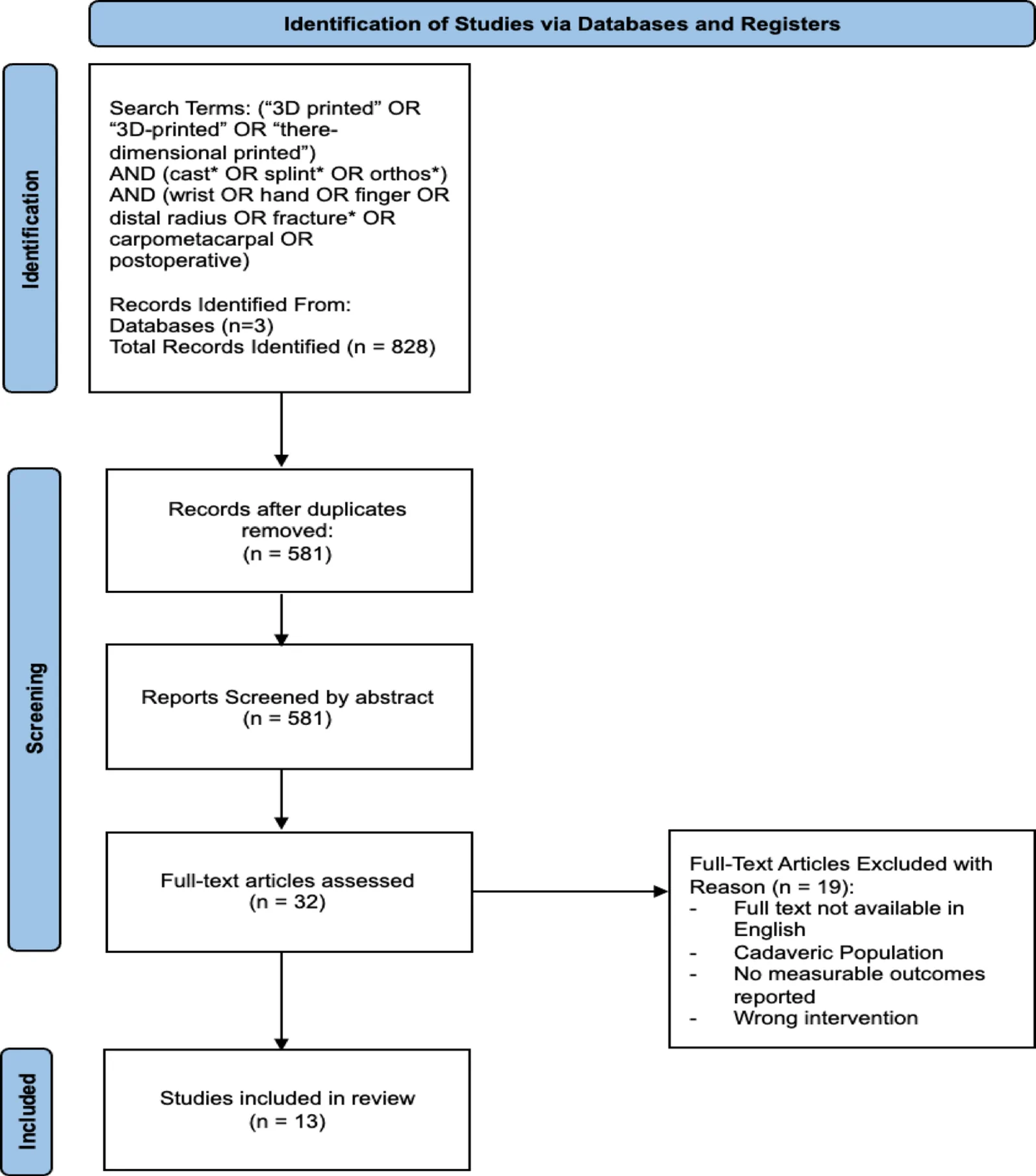
PRISMA diagram outlining the study selection process from initial search to final inclusion

Exclusion criteria included animal or in-vitro/bioengineering bench-only studies, case reports (n < 5), studies of 3D-printed internal implants or prosthetics unrelated to immobilization, reviews, editorials, opinion pieces, and conference abstracts without extractable outcome data (Fig. 1).

### Study selection, quality selection, and data extraction

Searches performed across three PubMed, Scopus, and Web of Science yielded a total of 828 studies. After removal of duplicates, 581 studies remained and were screened by title and abstract. Following screening, 32 full-text articles were assessed for eligibility. Of these, 19 studies were excluded for the following reasons: full text not available in English, cadaveric population, absence of measurable outcomes, or incorrect intervention. Ultimately, 13 studies met the inclusion criteria and were included in the final review, and screened for evaluation of 3D-printed casts, splints, or orthoses across fracture conditions, with both pediatric and adult populations represented. Comparators typically involved fiberglass casts, plaster casts, thermoplastic splints, or polymer orthoses, although it was not listed in some studies.

Two reviewers independently extracted data using a standardized approach and documented in a standardized Microsoft Excel sheet to capture study characteristics, methodological design, population demographics, intervention and comparator details, primary and secondary outcomes, adverse events, and dropout rates. Any disagreements were resolved through adjudication by a third reviewer.

Primary outcomes were defined as fracture healing or union, complication rates (malunion, nonunion, or surgery required for stability or healing), and time-to-fracture healing. Secondary outcomes included pain, patient satisfaction, comfort, function and range of motion (ROM), time to return to function, adverse skin events, hygiene, device weight, cost and production time, imaging interference, and resource utilization. Study characteristics included design, level of evidence, population summary, sample size, 3D-printed device intervention, comparator, and follow-up duration.

Risk of Bias was assessed independently by two reviewers using validated tools appropriate for each study design: Cochrane RoB-2, ROBINS-I, JBI Critical Appraisal Tools, and JBI Case Series Checklist. Conflicts were resolved through consensus with a third independent reviewer.

### Qualitative and quantitative data analysis

To address heterogeneity in this review, descriptive narrative analysis was used along with result stratification by age group and device type within upper-extremity fracture patients for meaningful clinical insights.

A limited quantitative synthesis was performed on comparative studies only across three outcome domains: pain, patient satisfaction, and functional outcomes. Continuous data were extracted as means and standard deviations when feasible. When multiple measures were reported within a single study for a given domain, the most clinically relevant outcome was selected to avoid double-counting. Functional outcomes included validated upper extremity scores (e.g., DASH and Mayo Wrist Score), and pain outcomes included VAS and FPS-R scales.

Effect sizes were calculated using standardized mean differences (SMD) represented in Hedges g values with 95% confidence intervals to account for variability in measurement scales.

Random-effects models using restricted maximum likelihood (REML) estimation were applied. The Knapp-Hartung method was used to adjust standard errors and confidence intervals for small-sample inference, and statistical significance was determined based on confidence intervals. Directionality was standardized so that positive effect sizes consistently favored 3D immobilization, with reverse coding applied where lower scores indicated better outcomes.

Studies lacking sufficient complete comparative statistics (e.g., medians without dispersion measures) and feasibility studies were excluded from quantitative pooling and summarized narratively. Given the limited number of included studies and observed heterogeneity, functional, pain, and patient satisfaction outcomes were analyzed quantitatively without accompanying forest plots. All analyses were conducted in R using the metafor package.

## Results

### Study characteristics

A total of 13 studies evaluating 3D-printed immobilization devices for limb fractures were included (Table 1). 12 included studies evaluated immobilization for upper-extremity fractures. Only one study evaluated non-upper-extremity fractures; therefore, it was excluded from the final analysis because insufficient data was available to support a meaningful clinical insight and comparison of anatomical sites [19].

**Table 1:** Overview of study design, populations, interventions, comparators, and follow‑up across included studies

References	Study design	Level of evidence	Population age group	Sample size (3D vs control)	Fracture type	Intervention	Comparator	Follow-up duration
Chen [25]	Pilot comparative study	Level II	Adults	60 (3D:20, Plaster:20, Splint:20)	Forearm fractures	Cast (3D-printed external orthopedic cast)	Comparator 1: Traditional plaster external fixation (plaster cast) Comparator 2: Splint external fixation (splinting)	Not explicitly stated, post-treatment clinical assessment
Skibicki [21]	Randomized controlled trial	Level II	Pediatric	23 limbs (3D:11, Fiberglass:12)	Wrist or forearm fractures	3D-printed orthosis for fracture immobilization	Conventional fiberglass cast (traditional cast padding overwrapped with a fiberglass roll)	Not explicitly stated, assessed until radiographic healing
Guebeli [11]	Randomized controlled trial	Level I	Adults	39 (3D:19, Fiberglass:20)	Minimally displaced or non-displaced distal radial fractures	Splint (Patient-specific, three-quarter circumferential forearm splint with a dorsoulnar longitudinal opening and loops for fixation, manufactured using digital light processing [DLP] technology and photosensitive photopolymer resin)	Conventional fiberglass cast	1 year
Tobler-Ammann [22]	Randomized feasibility trial	Level II-III	Adults	19 (3D:10, Fiberglass:9)	Distal radius or scaphoid fractures	Orthosis (Patient-specific, open-design 3D-printed wrist orthosis made from polyamide and fixed using hook and loop/Velcro straps)	Traditional fiberglass cast	6–8 weeks
Chen [26]	Prospective clinical trial	Level IV	Adults and Pediatrics	10 (3D only)	Distal radius fractures	Cast (Patient-specific short arm orthopedic cast split longitudinally into two halves, showcasing a holed/ventilated web pattern and secured via Velcro straps)	No control group	6 weeks
Surucu [27]	Prospective case series	Level IV	Adults and Pediatrics	24 (3D only)	Upper extremity fractures (distal radius fractures, metacarpal fractures, humeral shaft fractures, forearm fractures, proximal phalanx fractures)	Cast (Patient-specific 3D-printed medical cast made from polyethylene terephthalate glycol [PETG] with a parameter-optimized Voronoi design)	No control group	6–18 months
Xiao [8]	Prospective Comparative study	Level II-III	Adults	40 (3D:20, Polymer:20)	Colles fracture	Cast (Instant 3D-printed wrist cast)	Polymer orthosis	12 weeks
Ma [7]	Multicenter randomized clinical trial (open-label, analyst-blinded)	Level I	Adults	110 randomized (3D: 54, Fiberglass: 56)	Distal radius fractures	Splint (Prefabricated, topology-optimized nylon/polyamide splint)	Traditional cast immobilization	12 weeks (6-week immobilization + 6-week observation)
Khoury [23]	Randomized clinical trial with crossover sub-study	Level II	Adults	34 (3D: 18, control: 16) Crossover subset: 9 patients	Distal radius fractures	Splint (3D-printed custom polyolefin/polypropylene splint)	Conventional removable splint (CRS); traditional cast (contextual comparison)	Short-term follow-up during immobilization period (exact duration not specified)
Xu [24]	Single-Centre randomized clinical trial (exploratory)	Level II	Pediatric	56 (3D: 28, Control: 28)	Distal radius/forearm fractures	Splint (Customized 3D-printed splint optimized via finite element analysis)	Traditional U-shaped forearm plaster cast immobilization	Until external fixation removal (short-term follow-up)
Factor [28]	Prospective single-arm feasibility study	Level IV	Adults	20 (3D only)	Wrist and hand fractures (Distal radius fracture: 13, Scaphoid fracture: 4, Hamate fracture: 2, Base of first metacarpal fracture: 1)	Cast (Patient-specific 3D-printed orthopedic forearm cast / thumb spica cast)	No control group (standard plaster used initially, then converted)	~ 2, 6, and 12 weeks
Lazzeri [13]	Prospective case series (pre-marketing safety study)	Level IV	Pediatric	10 (3D only)	Distal radius buckle fractures	Cast (Patient-specific 3D-printed orthopedic forearm cast consisting of two shells made of acrylonitrile butadiene styrene [ABS] with ventilation holes and plastic ties)	No control group	21 days (three evaluations within first 3 weeks)

Randomized clinical trials accounted for 4 studies (33.33%), while pilot and feasibility trials also accounted for 4 studies (33.33%). Additionally, there were 2 non-randomized comparative studies (16.67%) and 2 single-arm case series (16.67%).

The included studies comprised a total of 456 participants, with comparative designs accounting for 392 participants, whereas single-arm studies contributed 64 participants. The included studies were generally small, with sample sizes ranging from 10 to 110 participants and a median sample size of approximately 32. Half of the studies (6/12) enrolled 25 or fewer participants.

Following the exclusion of the lower-extremity study, the remaining 12 upper-extremity trials primarily evaluated adults, with 7 studies representing 58.3% of the cohort. Pediatric-only populations were examined in 3 studies (25.0%), while 2 studies (16.7%) included a mixed population of both adult and pediatric participants. Across these 12 upper-extremity evaluations, 3D casts were the most frequently evaluated intervention, appearing in 6 studies (50.0%). This was followed by 3D splints, which were assessed in 4 studies (33.3%), while 3D orthoses were investigated in 2 studies (16.7%). Fractures specifically localized to the distal radius, forearm, and wrist accounted for 91.7% of the evaluated clinical fracture profiles.

### Risk of bias (RoB) and evidence assessment

The majority of the evidence base consisted of moderate-quality comparative studies, and no included study was found to be at low risk of bias (Table 2). Level II evidence represented the largest proportion of included studies, accounting for 4 of 12 studies (33.3%), followed by Level IV studies (4 of 12; 33.3%), Level II–III studies (2 of 12; 16.7%), and Level I randomized trials (2 of 12; 16.7%).

**Table 2:** Risk of bias (RoB) summary

References	Tool used	Overall judgement
Chen [25]	ROBINS-I	Moderate risk
Skibicki [21]	Cochrane RoB 2	Some concerns
Guebeli, [11]	Cochrane RoB 2	Some concerns
Tobler-Ammann [22]	Cochrane RoB 2	Some concerns
Chen [26]	ROBINS-I	Serious
Surucu [27]	JBI critical appraisal tools	Moderate risk
Xiao [8]	ROBINS-I	Moderate risk
Ma [7]	Cochrane RoB 2	Some concerns
Khoury [23]	Cochrane RoB 2	High risk
Xu [24]	Cochrane RoB 2	High risk
Factor [28]	JBI critical appraisal tools	Moderate risk
Lazzeri [13]	JBI case series checklist	Moderate risk

Randomized controlled trials accounted for 6 of 12 studies (50.0%). Among these, 4 of 6 studies (66.7%) were judged as having some concerns for overall risk of bias under Cochrane RoB 2, including Skibicki, Guebeli, Tobler-Ammann, and Ma [7, 11, 21, 22]. The remaining two randomized studies, Khoury and Xu, were judged to have a high risk of bias due to lack of blinding, deviations from intended interventions, and incomplete outcome reporting [23, 24].

Non-randomized comparative studies accounted for 3 of 12 studies (25.0%) and were evaluated using ROBINS-I. Chen 2020 and Xiao 2024 [8, 25, 26] demonstrated moderate risk of bias, while Chen 2017 was judged to have serious risk of bias due to its single-arm design and limited control of confounding variables.

Feasibility and case-series studies accounted for 3 of 12 studies (25.0%) and were assessed using JBI appraisal tools. Surucu, Factor, and Lazzeri [13, 27, 28] were all judged to have a moderate risk of bias, reflecting the inherent limitations of uncontrolled observational designs.

Overall, the majority of included studies fell within the moderate-to-high risk of bias range, with common methodological limitations including small sample sizes, lack of blinding, heterogeneous outcome reporting, and limited long-term follow-up.

### Primary outcomes

**Fig. 2:**
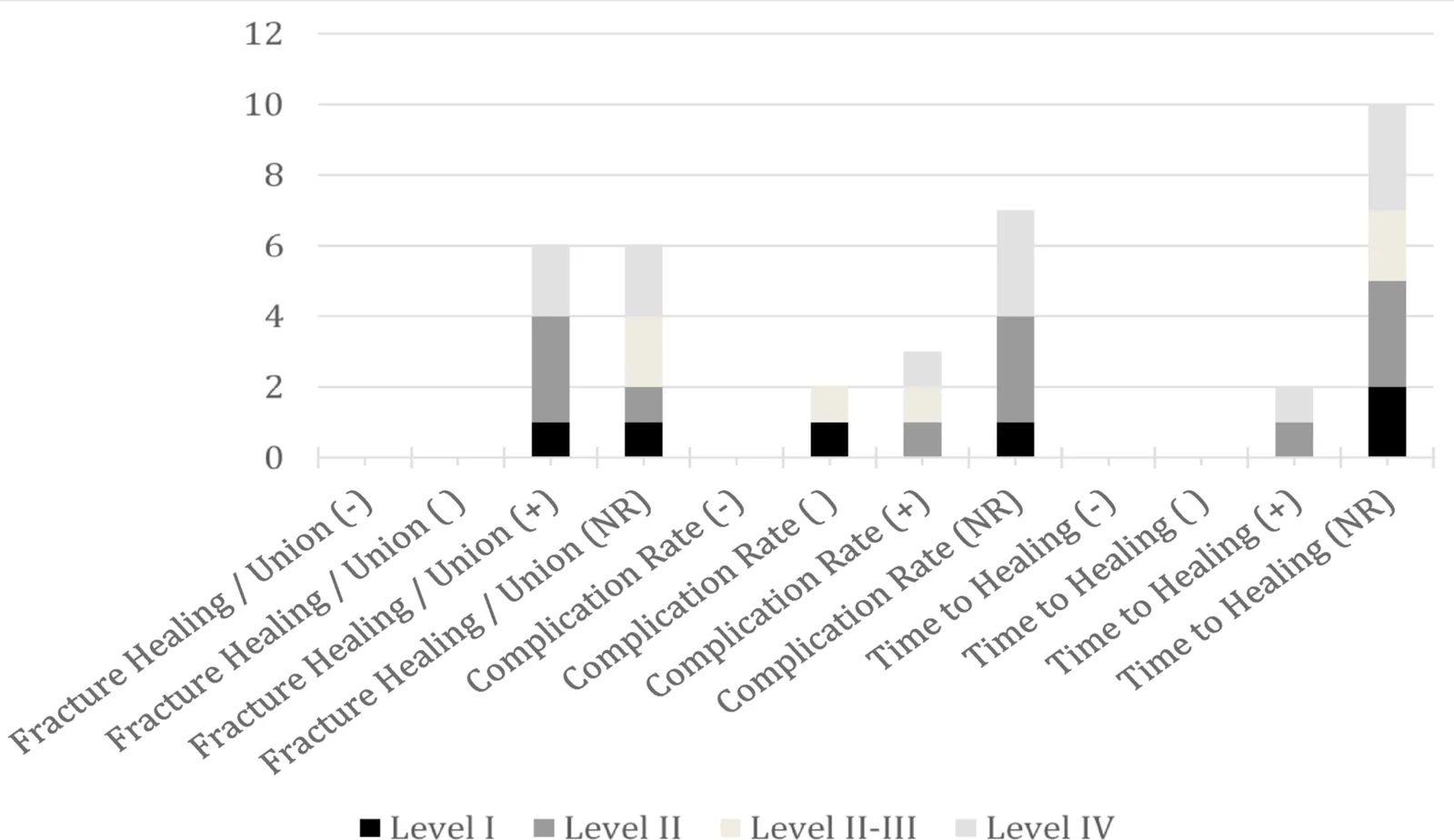
Distribution of primary study outcomes categorized as negative, neutral, or positive toward 3D-printed casts across four evaluation domains

Fracture healing outcomes uniformly favored 3D-printed immobilization across low-to-high quality evidence (Levels I-IV), with 100% union or treatment success reported in all studies where healing was explicitly assessed, regardless of device type; complications showed heterogeneity across studies. The strength of evidence was low-to-moderate, driven primarily by prospective comparative and feasibility studies with variable complication reporting (Table 3, Fig. 2).

**Table 3:** Primary outcomes for fracture healing, complication rates, and time to healing across included studies of 3D‑printed immobilization devices

References	Fracture type	Comparator	Level of evidence	Fracture healing/union	Complication rate (mal/non-union, operation)	Time to healing	Overall direction
Chen [25]	Forearm fractures: Colles (46), Smith (12), ulnoradial diaphyseal (2)	Plaster cast (n = 20) and splint fixation (n = 20)	Level II	20/20 (100%) achieved treatment goal; no secondary reduction, for all groups	Exact rate Not Reported; 0 cast breakage reported	6 weeks immobilization; function assessed at 3 months	Favors 3D for combined efficacy and comfort
Skibicki [21]	Acute nondisplaced/minimally displaced pediatric wrist or forearm fractures; distal radius Salter-Harris I, buckle metaphyseal fractures, ulnar fractures	Conventional fiberglass cast	Level II	Fully healed radiographically: 11/11 (100%)	1 broken cast replacement; no treatment crossover/major complications	Mostly 4 weeks; some followed to 8 weeks	Comparable healing; comfort/satisfaction favors 3D
Guebeli [11]	Non-displaced or minimally displaced distal radius fractures	Conventional fiberglass cast	Level I	19/19 (100%) radiological healing in 3D group; all patients healed overall	Visit 5 (6 weeks): 8/19 (42.1%) in 3D group versus 4/18 (22.2%) in cast group; severe complication only in cast group (1 fracture displacement)	Immobilization 6 weeks; follow-up to 52 weeks	Comparable clinical effectiveness; satisfaction favors 3D
Tobler-Ammann [22]	Distal radius or scaphoid fractures	Conventional fiberglass cast	Level II-III	Equivalent; no displacement; one cast-treated scaphoid nonunion requiring surgery	Surgery in cast group (1 case); none in 3D group	Not reported	Favors 3D for experience; healing equivalent with fewer serious complications
Chen [26]	Distal radius fractures (6 distal radius + ulnar styloid, 3 isolated distal radius, 1 distal radius + ulna)	No Control Group	Level IV	10/10 (100%) maintained reduction; no loss of reduction	1/10 transient blister (10%); 0/10 pressure sores; 0/10 compartment syndrome; 0/10 cast breakage	7-week follow-up; time not explicitly stated	Favors 3D for satisfaction and safety
Surucu [27]	Upper extremity fractures (distal radius fractures, metacarpal fractures, humeral shaft fractures, forearm fractures, proximal phalanx fractures)	No Control Group	Level IV	Union achieved in all fractures (100%)	No major complications reported	Union in all fractures within 4–6 weeks	Demonstrates effectiveness and high satisfaction
Xiao [8]	Colles fracture	Polymer orthosis	Level II-III	Better maintenance of reduction at 2 and 12 weeks in 3D	2 control group patients experienced skin irritation	Not directly reported; 2–12 week outcomes tracked	Favors 3D for immobilization, pain, function, and safety
Ma [7]	Acute closed distal radius fractures (DRFs), classified as type A and type B fractures according to the AO Foundation/Orthopaedic Trauma Association (AO/OTA) classification	Traditional cast immobilization	Level I	Radiographic alignment similar; wrist function better early with 3D	Shoulder-elbow pain and dysfunction (3D:4 patients; Control: 15 patients) Skin irritation (3D:15 patients; Control:21 patients)	Not reported	Favors 3D for early function and pain
Khoury [23]	Distal radius fractures	Conventional removable splint (CRS); traditional cast (contextual comparison)	Level II	Not reported	Pressure pain and sores slightly higher in 3D (3D: 33%/28% vs Control: 6%/6%)	Not reported	Similar subjective outcomes; adverse skin events higher in 3D
Xu [24]	Distal radius/forearm fractures	Traditional U-shaped forearm plaster immobilization	Level II	28/28 achieved union by week 4 (100%)	3D: 7.1% (2 cases of joint dysfunction presenting as limited flexion, which resolved after 4 weeks of rehabilitation) Control: 10.7% (3 cases of skin irritation at the ulnar styloid process, which resolved within 2 weeks after cast removal)	4 weeks to union for all fractures in study	Favors 3D for pain, satisfaction, and Mayo score; higher cost/time
Factor [28]	Wrist and hand fractures (Distal radius fracture: 13, Scaphoid fracture: 4, Hamate fracture: 2, Base of first metacarpal fracture: 1)	No Control Group (standard plaster used initially, then converted)	Level IV	20/20 radiographic union at final follow up (100%)	15% (3 cases of cast breakage out of 20 patients)	3 months to union	Shows feasibility and effective immobilization
Lazzeri [13]	Distal radius buckle fractures	No control group	Level IV	10/10 fully resolved tenderness over fracture site (100%)	0 significant skin lesions; 20% minor irritation which resolved completely, 0% mechanical complications	Not reported	Favors 3D for comfort and feasibility

#### Stratification by population

Adult-only studies accounted for 6 of 12 studies (50%). Fracture healing outcomes were consistently high across all adult comparative and feasibility studies, with approximately 100% reported union or treatment success, and no evidence of nonunion attributable to 3D-printed immobilization [11, 22, 24, 25]. Complication reporting was variable but major adverse events were generally low, with isolated cases of surgical intervention or fracture displacement occurring in comparator groups rather than 3D device groups [11, 22]. Minor skin irritation and pressure-related symptoms were inconsistently distributed, with some 3D groups reporting slightly higher rates (Khoury 2022: pressure pain 33% vs 6%; sores 28% vs 6%) while others demonstrated fewer skin-related complications compared with conventional casting (Ma 2024: skin irritation 15 vs 21 patients) [7, 23].

Pediatric-only studies accounted for 2 of 12 studies (16.7%). Healing outcomes were uniformly favorable, with Skibicki 2021 reporting 100% radiographic healing (11/11 limbs) and Lazzeri 2022 describing clinically satisfactory healing without treatment failures [13, 21]. Complication rates in pediatric cohorts were minimal, limited to isolated minor skin irritation and one case of cast breakage in a fiberglass comparator group; no serious adverse events including nonunion, compartment syndrome, or loss of reduction, were reported in either pediatric study. The direction of effect was consistent with adult cohorts, though the limited number of pediatric studies precludes formal cross-population comparison.

Mixed or unspecified population studies accounted for 4 of 12 studies (33.3%) [7, 8, 27, 28]. These studies generally supported effective immobilization and high union rates; however, age-stratified outcomes were not consistently reported, limiting quantitative pooling and interpretation across subgroups.

#### Stratification by device type

Splint- and orthosis-based systems accounted for 5 of 12 studies, with healing outcomes uniformly favorable and no treatment failures attributable to 3D device design. Complication profiles were generally favorable, with fewer skin-related adverse events compared with conventional splints or casts in most studies, though Khoury [7, 23, 24] represented an exception with slightly higher rates of pressure pain and sores in the 3D splint group.

Cast-based systems accounted for 4 of 12 studies, with 100% union or treatment success reported across all studies where healing was explicitly assessed [8, 11, 21, 25]. Major complications were absent or confined to comparator groups, including one case of fracture displacement and one scaphoid nonunion requiring surgery, both occurring in conventional cast groups [11, 22]. Feasibility studies without comparator groups reported uniformly high union rates and low complication rates, with only isolated minor skin irritation or cast breakage, supporting the safety profile of 3D-printed immobilization across device designs [13, 26–28]. Across device types, no meaningful differential in healing success was identified between splint- and cast-based 3D systems; heterogeneity in complication reporting, particularly for minor skin events, was present across both device categories.

### Secondary outcomes

**Fig. 3:**
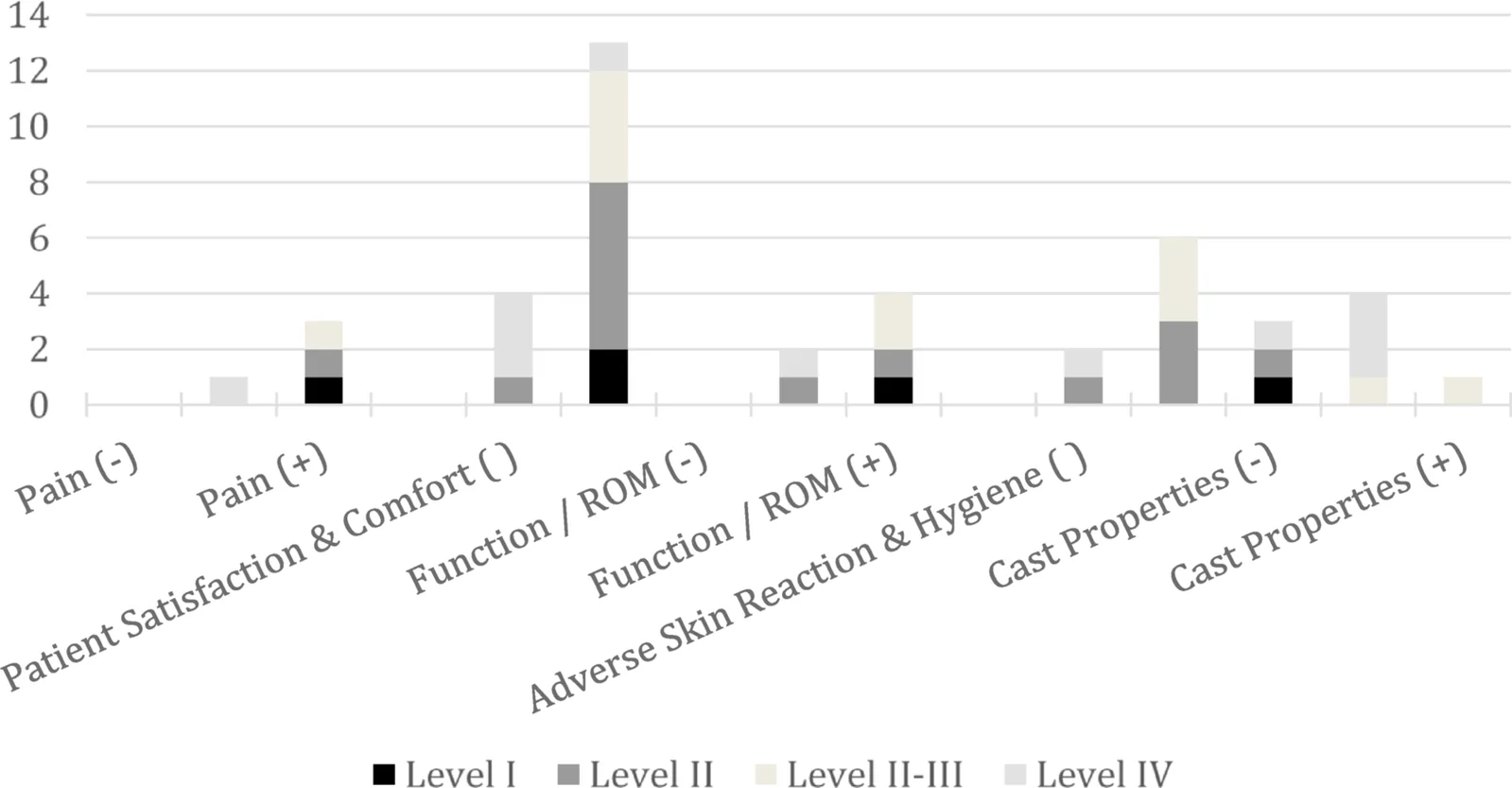
Distribution of secondary study outcomes categorized as negative, neutral, or positive toward 3D‑printed casts across domains

Secondary outcomes were defined as follows: pain, patient satisfaction, comfort, function/range of motion (ROM), time to return to function, adverse skin events, hygiene, device properties (device weight, cost, and time to produce, imaging interference, and resource use) (Table 4, Fig. 3).

**Table 4:** Evidence summary for secondary outcomes, showing study counts and direction of findings across key domains

References	Level of evidence	Sample size	Domain	Measure/scale	3D result	Comparator result	Difference/effect	Direction
*Pain*								
Xiao [8]	Level II-III	40	Pain	VAS pain at 2 weeks & 6 weeks	1.6 ± 0.48 (2 weeks) 0.69 ± 0.32 (6 weeks)	1.8 ± 0.38 (2 weeks) 0.74 ± 0.33 (6 weeks)	Significant at 2 weeks (*p* = 0.033) Not significant at 6 weeks (*p* = 0.755)	Favors 3D
Ma [7]	Level I	110	Pain	VAS pain median (IQR)	2 (2 to 3) at 2 weeks 1 (1 to 2) at 6 weeks	3 (2 to 4) at 2 weeks 2 (1 to 3) at 6 weeks	*p* = 0.03 (2 weeks) *p* = 0.03 (6 weeks)	Favors 3D early
Xu [24]	Level II	56	Pain	Wong-baker faces pain scale-revised (FPS-R)	3.18 ± 1.79	4.00 ± 1.30	*p* = 0.049	Favors 3D
Factor [28]	Level IV	20	Pain	VAS pain at 2 and 6 weeks	0.9 ± 1.1 (2 weeks) 0.6 ± 1.0 (6 weeks)	N/A	Improved within-group	Supports effectiveness
Guebeli [11]	Level I	39	Pain	VAS at rest and under load	No significant difference	No significant difference	N/A	N/A
*Patient satisfaction*								
Chen [25]	Level II	60	Patient satisfaction	12-point questionnaire	8.65 ± 1.04	Plaster: 6.85 ± 1.14; Splint: 8.10 ± 1.25	*p* < 0.001	Favors 3D
Guebeli [11]	Level I	39	Patient satisfaction	Modified 12-point questionnaire	9.8 at 6 weeks	8.7 at 6 weeks	Difference + 1.1 favoring 3D	Favors 3D (non-inferior)
Tobler-Ammann [22]	Level II-III	19	Patient satisfaction	PRSEQ score	8.4 ± 1.6	5.6 ± 2.7	*p* < 0.001–0.01	Favors 3D
Chen [26]	Level IV	10	Patient Satisfaction	Satisfaction (15-point)	Mean 11.5/15; 100% prefer 3D	Preference versus plaster: 0%	Descriptive	Favors 3D
Surucu [27]	Level IV	24	Patient satisfaction	QUEST 2.0	Mean 4.7 (high)	N/A	Descriptive	High satisfaction
Xiao [8]	Level II-III	40	Patient satisfaction	Immobilization effectiveness score at 6 weeks	9.8 ± 0.96	9.4 ± 1.07	*p* < 0.001	Favors 3D
Khoury [23]	Level II	34	Patient satisfaction	Subjective outcome questionnaire	86.25 ± 8.00	85.18 ± 9.49	*p* = 0.932	No difference
Khoury [23]	Level II	34	Patient satisfaction	SF-mental component summary	51.86 ± 7.82	45.57 ± 5.55	*p* = 0.041	Favors 3D
Xu [24]	Level II	56	Patient Satisfaction	Patient satisfaction questionnaire	2.71 ± 1.27	1.57 ± 0.84	*p* = 0.004	Favors 3D
Skibicki [21]	Level II	23	Patient Satisfaction	Satisfaction VAS 0–10	9.4/10	7.2/10	*p* = 0.009	Favors 3D
Factor [28]	Level IV	20	Patient satisfaction	Patient questionnaire	95% of patients “totally agree”	N/A	Descriptive	High satisfaction
Lazzeri [13]	Level IV	10	Patient satisfaction	Likert (child/parent)	Child: 4.7 ± 0.1; Parent: 4.2 ± 0.3	N/A	Descriptive	High satisfaction
*Comfort*								
Skibicki [21]	Level II	23	Comfort	VAS comfort	9.1	7.1	*p* = 0.02	Favors 3D
Tobler-Ammann [22]	Level II-III	19	Comfort	Perceived comfort	Less discomfort by week 4	More discomfort	*p* = 0.03	Favors 3D
Xiao [8]	Level II-III	40	Comfort	Immobilization Satisfaction score at 6 weeks	11.6 ± 1.4	10.8 ± 1.8	*p* < 0.001	Favors 3D
Chen [26]	Level IV	10	Comfort	0–3 item score	3.0/3.0 (full marks)	Not reported	N/A	N/A
Surucu et al. [27]	Level IV	24	Comfort	QUEST 2.0 comfort item	4.9/5	N/A	N/A	N/A
Ma [7]	Level I	110	Comfort	Swelling degree Scale at 2 weeks	1 (0 to 1)	1 (1 to 2)	*p* = 0.02	Favors 3D
Xu [24]	Level II	56	Comfort	Outcome questionnaire	2.39 ± 1.13	1.61 ± 0.74	*p* < 0.05	Favors 3D
*Function and range of motion (ROM)*								
Xiao [8]	Level II-III	40	Function/ROM	DASH	20.2 ± 5.7 (2 weeks) 8.1 ± 3.9 (6 weeks)	22.6 ± 5.7 (2 weeks) 10.2 ± 3.1 (6 weeks)	*p* < 0.001 (2 weeks & 6 weeks)	Favors 3D
Ma [7]	Level I	110	Function/ROM	Gartland-Werley; ROM	Median of 30° (IQR, 24° to 45°)	Median of 22° (IQR, 18° to 30°)	*p* < 0.001	Favors 3D early
Xu [24]	Level II	56	Function/ROM	Mayo wrist score	89.64 ± 8.71	88.57 ± 6.22	*p* = 0.044	Favors 3D (modest)
Chen [25]	Level II	60	Function/ROM	Cooney modification of Green & O’Brien score	80% excellent/good	Plaster: 65%; Splint: 70%	N/A	N/A
Guebeli [11]	Level I	39	Function/ROM	ROM, grip strength, DASH, PRWE	No apparent differences at 12, 26, 52 weeks	No apparent differences	N/A	N/A
Khoury [23]	Level II	34	Function/ROM	DASH	45.17 ± 20.57	37.50 ± 19.21	*p* = 0.336	No difference
Factor [28]	Level IV	20	Function/ROM	DASH	18.7 ± 9.5 (2 weeks) 7.6 ± 7.6 (6 weeks)	N/A	Favors functional capability	Supports effectiveness
Tobler-Ammann [22]	Level II-III	19	Function/ROM	Routine activities	Fewer difficulties by weeks 2 & 6	More difficulties	*p* = 0.033–0.034	Favors 3D
*Adverse skin reaction*								
Tobler-Ammann [22]	Level II-III	19	Adverse skin reaction	Sweating, irritation, pressure complaints	Generally lower	Higher	Moderate-to-large effect sizes	Favors 3D
Xiao [8]	Level II-III	40	Adverse skin reaction	Skin irritation	0 patients	2 patients	Observed difference	Favors 3D
Khoury [23]	Level II	34	Adverse skin reaction	Pressure pain/sores	33% pain; 28% sores	6% pain; 6% sores	Not significantly significant	No difference
Skibicki [21]	Level II	23	Adverse skin reaction	Patient-reported skin irritation categories	Skin intact: 8/11; minor irritation: 2/11; substantial irritation: 1/11	Skin intact: 2/12; minor irritation: 9/12; substantial irritation: 1/12	*p* = 0.01	Favors 3D
Lazzeri [13]	Level IV	10	Adverse skin reaction	Likert (child/parent)	Child: 2.2 ± 1.0; Parents: 1.5 ± 0.6	N/A	Descriptive	High satisfaction
*Hygiene*								
Chen [25]	Level II	60	Hygiene	Ventilation/odor/itch	Cast odor / smell 1.90 ± 0.718, Skin itchiness 2.20 ± 0.523	Plaster cast odor/smell 1.50 ± 0.513, Splint fixation: cast odor/smell 2.05 ± 0.510. Plaster cast itch 1.80 ± 0.410, Splint fixation itch 2.35 ± 0.587	*p* < 0.05	Favors 3D
Skibicki [21]	Level II	23	Hygiene	Cast care burden categories	No hassle: 10/11; minimal burden: 1/11	No hassle: 2/12; minimal burden: 6/12; moderate burden: 3/12; very difficult: 1/12	*p* = 0.0006	Favors 3D
Xiao [8]	Level II-III	40	Hygiene	Ease of hygiene/inspection	Improved	Worse	Significant	Favors 3D
*Cast properties*								
Xiao [8]	Level II-III	40	Device weight	Patient report	Lighter weight	Heavier	Significant (part of satisfaction)	Favors 3D
Guebeli [11]	Level I	39	Device weight	Measured weight	111 g average	Approximately > 333 g (3D was less than one-third of cast weight)	N/A	N/A
Surucu et al. [27]	Level IV	24	Device weight	QUEST 2.0 weight item	4.7/5	N/A	N/A	N/A
Xiao [8]	Level II-III	40	Time	Cost & time	~ 25 min	Not Reported	Observed	Feasible
Xu [24]	Level II	56	Cost	Cost & time	362.21 ± 13.81 CNY; 2.85 ± 0.93 h	248 CNY; 0.72 ± 0.34 h	*p* = 0.001	Favors Control
Lazzeri [13]	Level IV	10	Cost	Production cost	EUR 30 per cast	N/A	Descriptive	Feasible
Lazzeri [13]	Level IV	10	Cost/time	Supply chain delivery time	10/10 devices manufacture within 24–48 h	N/A	Descriptive	Feasible
Factor [28]	Level IV	20	Cost/time	In-hospital production time	161 ± 8 min (146–182)	N/A	Workflow feasibility	Feasible
Guebeli [11]	Level I	39	Cost	Material cost	US$20 per DLP splint; US$6 if produced with FDM	N/A	Moderate material cost	N/A
Guebeli [11]	Level I	39	Time	Time from scan to finished splint	145 min average	Cast application ~ 30 min	Conventional cast faster	Favors conventional
Surucu et al. [27]	Level IV	24	Cost	Device production cost	US$167–223	Conventional casts significantly cheaper	N/A	Favors conventional

### Pain outcomes

Pain outcomes generally favored 3D-printed immobilization across moderate-quality evidence (Levels I–IV), with a small-to-moderate reduction in early pain scores most consistently demonstrated in splint-based systems; cast-based systems showed no significant difference. The strength of evidence was moderate, driven primarily by randomized and prospective comparative studies.

#### Stratification by population

Adult-only studies accounted for 4 of 5 studies (80%). Pain reduction was greater and more consistent in adult populations, most clearly supported by higher-level evidence. The multicenter randomized clinical trial by Ma (Level I) demonstrated a statistically significant but modest reduction in pain at both 2 and 6 weeks with 3D-printed splints (median VAS 2 vs 3 at 2 weeks; *p* = 0.03), indicating a small absolute but consistent early effect [7]. The prospective comparative study by Xiao (Level II–III) similarly showed a small reduction in early pain scores at 2 weeks (1.6 ± 0.48 vs 1.8 ± 0.38; *p* = 0.033), with no sustained difference at 6 weeks, suggesting a transient early analgesic effect [8].

Pediatric-only studies accounted for 1 of 5 studies (20%), limiting the certainty of cross-age comparison. The pediatric randomized study by Xu (Level II) demonstrated a modest but statistically significant reduction in pain scores using the Wong-Baker scale (3.18 ± 1.79 vs 4.00 ± 1.30; *p* = 0.049), supporting a similar direction of effect, though based on low-to-moderate certainty evidence [24].

#### Stratification by device type

Splint-based systems accounted for 3 of 5 studies (60%) and demonstrated greater and more consistent pain reduction than cast-based interventions. Cast-based interventions accounted for 2 of 5 studies (40%), with more variable findings. Notably, the randomized controlled trial by Guebeli (Level I) found no significant differences in pain outcomes between 3D splints and conventional fiberglass casts, indicating that the effect magnitude may depend on device design, rigidity, or immobilization approach despite high-level evidence [11]. The feasibility study by Factor (Level IV) reported within-group reductions in pain over time but without comparator data, providing very low-certainty supportive evidence only for feasibility, not effect magnitude [28].

#### Meta-analysis

A random-effects meta-analysis utilizing REML estimation and Knapp–Hartung adjustments was conducted across two independent study arms (k = 2) evaluating patient pain levels. The pooled SMD indicated a small-to-moderate effect size favoring 3D immobilization over traditional casting methods (Hedges’ g = 0.36, 95% CI [− 1.94, 2.66]); however, this difference was not statistically significant (*p* = 0.295). There was a complete absence of observed between-study heterogeneity (*p* = 0.380; I^2^ = 0.00%). These findings suggest a stable trend toward improved pain management with 3D-printed devices; however, the small sample size results in a wide confidence interval spanning zero.

### Patient satisfaction and comfort

Patient satisfaction consistently favored 3D-printed immobilization across moderate-to-high quality evidence (Levels I–IV), with significant improvements demonstrated in both splint- and cast-based systems across adult and pediatric populations. The strength of evidence was moderate-to-high, driven primarily by randomized controlled trials and prospective comparative studies.

#### Stratification by population

Adult-only studies accounted for 7 of 11 studies (63.6%), with 6 of 7 (85.7%) favoring 3D-printed immobilization and 1 of 7 (14.3%) showing no difference. In the Level II comparative study by Chen (n = 60), patient satisfaction scores were significantly higher in the 3D-printed cast group compared with plaster immobilization (8.65 ± 1.04 vs 6.85 ± 1.14, *p* < 0.001) [25]. The randomized controlled trial by Guebeli (Level I; n = 39) demonstrated higher satisfaction with 3D-printed splints compared with fiberglass casts (9.8 vs 8.7 at 6 weeks), a mean improvement of + 1.1 points [11]. The prospective comparative study by Xiao also demonstrated higher immobilization effectiveness scores with 3D casts (9.8 ± 0.96 vs 9.4 ± 1.07; *p* < 0.001) [8]. The crossover study by Khoury (n = 34) showed no significant difference in subjective outcome scores (86.25 ± 8.00 vs 85.18 ± 9.49; *p* = 0.932), although mental health-related quality-of-life scores (SF-MCS) favored 3D-printed splints (51.86 ± 7.82 vs 45.57 ± 5.55; *p* = 0.041) [23]. Additional adult feasibility studies reported uniformly high satisfaction with 3D-printed devices, though these lacked comparator groups.

Pediatric-only studies accounted for 3 of 11 studies (27.3%), with all reporting high satisfaction favoring 3D-printed immobilization. In the randomized controlled trial by Skibicki (n = 23 limbs), satisfaction scores were significantly higher in the 3D orthosis group compared with fiberglass casting (9.4/10 vs 7.2/10, *p* = 0.009) [21]. Xu et al. [24] (n = 56) also demonstrated significantly higher satisfaction scores with customized 3D splints (2.71 ± 1.27 vs 1.57 ± 0.84, *p* = 0.004). In the pediatric feasibility study by Lazzeri [13], both child and parent Likert scores indicated very high satisfaction (child: 4.7 ± 0.1; parent: 4.2 ± 0.3), though no comparator group was included. Across both age groups, the direction of effect was consistently positive, with pediatric studies demonstrating numerically larger effect magnitudes, though cross-population comparisons remain limited by differing satisfaction scales.

#### Stratification by device type

Splint-based systems accounted for 5 of 11 studies, with 4 of 5 (80%) demonstrating significantly higher satisfaction compared with conventional immobilization. High-quality randomized trials, including Guebeli and Xu [11, 24], attributed improvements to enhanced comfort, fit, and usability. Cast-based interventions accounted for 4 of 11 studies, with 3 of 4 (75%) favoring 3D-printed casts, including significant improvements reported by Chen and Xiao [8, 25]. Orthosis-based designs generally contributed positively to patient satisfaction, particularly in pediatric populations. Across device types, the direction of effect was consistently favorable for 3D-printed immobilization, with no clear differential advantage identified between splint- and cast-based designs on satisfaction outcomes alone.

#### Meta-analysis

A random-effects meta-analysis using REML estimation with Knapp–Hartung adjustment was conducted across four independent study arms (k = 4). The pooled standardized mean difference demonstrated a large effect favoring 3D-printed immobilization over traditional methods (Hedges’ g = 0.83, 95% CI [− 0.21, 1.88]), although statistical significance was not reached (*p* = 0.085). Significant between-study heterogeneity was identified (*p* = 0.005; I^2^ = 77.50%). The wide confidence interval and resulting non-significant *p*-value likely reflect the conservative nature of the Knapp–Hartung adjustment in the setting of a small, pooled sample and substantial interstudy variability, partly attributable to the inclusion of both adult and pediatric cohorts and mixed device types.

### Function/ROM and time to return to function

Functional outcomes favored 3D-printed immobilization in the early post-immobilization period across moderate-quality evidence (Levels I–IV), most consistently in splint-based systems; both splint- and cast-based designs demonstrated no significant difference at longer follow-up intervals in higher-quality trials. The strength of evidence was moderate, driven primarily by randomized and prospective comparative studies.

#### Stratification by population

Adult-only studies accounted for 7 of 8 studies (87.5%), with 5 of 7 (71.4%) favoring 3D-printed immobilization and 2 of 7 (28.6%) demonstrating no significant difference. In the prospective comparative study by Xiao [8] (Level II–III; n = 40), DASH scores were significantly lower in the 3D-printed cast group at both 2 weeks (20.2 ± 5.7 vs 22.6 ± 5.7) and 6 weeks (8.1 ± 3.9 vs 10.2 ± 3.1; *p* < 0.001 for both), indicating improved early functional recovery. The multicenter randomized trial by Ma [7] (Level I; n = 110) demonstrated superior early range of motion in the 3D splint cohort (median ROM 30°, IQR 24°–45°, vs 22°, IQR 18°–30°; *p* < 0.001). The comparative study by Chen [25] (Level II; n = 60) demonstrated higher rates of excellent or good functional outcomes with 3D-printed casts (80%) compared with plaster (65%) and splint immobilization (70%). In contrast, the randomized trial by Guebeli (Level I; n = 39) found no significant differences in ROM, grip strength, DASH, or PRWE scores at 12-, 26-, or 52-week follow-up, and Khoury [11, 23] (Level II; n = 34) similarly demonstrated no significant difference in DASH scores between groups (45.17 ± 20.57 vs 37.50 ± 19.21; *p* = 0.336). Additional adult feasibility studies supported favorable functional recovery; Factor reported progressive DASH score improvements over time without comparator controls, and Tobler-Ammann [22, 28] demonstrated fewer difficulties with routine activities in the 3D orthosis group at both 2 and 6 weeks. Taken together, adult data suggest an early functional advantage with 3D-printed devices that attenuates at longer follow-up.

Pediatric-only studies accounted for 1 of 8 studies (12.5%). Xu [24] (Level II) demonstrated modest but statistically significant functional improvement in children treated with customized 3D splints compared with conventional plaster immobilization (Mayo Wrist Score: 89.64 ± 8.71 vs 88.57 ± 6.22; *p* = 0.044), consistent with the early benefit observed in adult cohorts, though cross-population inference remains limited by the single pediatric study.

#### Stratification by device type

Splint-based systems accounted for 4 of 8 studies (50%), with 3 of 4 (75%) favoring 3D-printed splints, particularly for early ROM recovery and functional performance. Cast-based interventions accounted for 3 of 8 studies (37.5%), with 2 of 3 (66.7%) favoring 3D-printed casts. Orthosis-based designs demonstrated favorable functional recovery and reduced difficulty with routine activities during early rehabilitation. Across device types, the early functional advantage was most clearly demonstrated in splint-based systems, while cast- and orthosis-based designs showed more variable results, particularly at extended follow-up.

#### Meta-analysis

A random-effects meta-analysis utilizing REML estimation and Knapp–Hartung adjustments was conducted across three independent study arms (k = 3) evaluating functional recovery and upper extremity range of motion. The pooled SMD indicated a negligible, non-significant difference between 3D immobilization and traditional control groups (Hedges’ g = 0.13, 95% CI [− 1.02, 1.28], *p* = 0.679). Moderate between-study heterogeneity was observed, though it did not meet the threshold for statistical significance (*p* = 0.128; I^2^ = 51.65%). These findings suggest that 3D-printed immobilization devices provide equivalent clinical utility to traditional plaster or splints regarding long-term functional performance and joint mobility, with early advantages not sustained at longer follow-up intervals.

### Adverse skin events and hygiene

Skin-related outcomes favored 3D-printed immobilization across moderate-quality evidence (Levels II–IV), with consistent reductions in irritation, pressure complaints, and hygiene burden demonstrated across both splint- and cast-based systems in adult and pediatric populations. The strength of evidence was moderate, driven primarily by prospective comparative and randomized studies.

#### Stratification by population

Adult-only studies accounted for 5 of 9 studies (55.6%). The Level II comparative study by Chen [25] (n = 60) demonstrated improved ventilation and reduced odor and itching with 3D-printed casts compared with plaster immobilization (*p* < 0.05). Tobler-Ammann [22] (Level II–III; n = 19) reported lower rates of sweating, irritation, and pressure complaints in patients treated with 3D orthoses, with moderate-to-large effect sizes favoring 3D immobilization. Xiao [8] (Level II–III; n = 40) reported no cases of skin irritation in the 3D cast cohort compared with 2 cases in the conventional immobilization group, while also demonstrating improved hygiene access and inspection capability. In contrast, Khoury [23] (Level II; n = 34) found no statistically significant difference in pressure pain or sores between groups, though reported rates of pressure pain (33% vs 6%) and sores (28% vs 6%) varied between cohorts.

Pediatric-only studies accounted for 3 of 9 studies (33.3%), all favoring 3D-printed immobilization. In the randomized study by Skibicki [21] (Level II; n = 23 limbs), intact skin was observed in 8 of 11 patients (72.7%) treated with 3D orthoses compared with 2 of 12 patients (16.7%) treated with fiberglass casts, while minor irritation was substantially more common in the conventional cast group (75.0% vs 18.2%; *p* = 0.01). Cast-care burden was also markedly lower in the 3D group, with 10 of 11 patients (90.9%) reporting “no hassle” compared with only 2 of 12 (16.7%) in the conventional cast group (*p* = 0.0006). The pediatric feasibility study by Lazzeri [13] reported favorable child and parent skin-related satisfaction scores, though no comparator group was included. Across populations, the magnitude of skin-related benefit appeared comparable between adult and pediatric cohorts, with pediatric studies demonstrating particularly pronounced reductions in cast-care burden.

#### Stratification by device type

Splint- and orthosis-based systems accounted for 4 of 9 studies (44.4%), with all comparative studies favoring 3D-printed devices for reducing irritation, sweating, pressure complaints, and cast-care burden. Cast-based interventions accounted for 3 of 9 studies (33.3%), with all studies demonstrating improved hygiene access, ventilation, or lower skin irritation rates compared with conventional casts. Across device types, open-lattice and ventilated 3D designs consistently demonstrated superior cutaneous tolerance and hygiene-related usability compared with traditional immobilization methods, with no evidence of differential advantage between splint- and cast-based 3D devices on skin outcomes. Overall, 7 of 9 studies (77.8%) favored 3D-printed immobilization, 1 (11.1%) demonstrated no significant difference, and 1 (11.1%) was descriptive without comparator data.

### Device properties

Cast properties data were heterogeneous and limited, and it has been categorized into device weight, cost, and production time.

#### Device weight

Device weight consistently favored 3D-printed immobilization systems. In two comparative studies with quantitative reporting, 3D devices were approximately 65–70% lighter than conventional casts, with one study reporting an average weight of 111 g for 3D devices versus ~ 333 g for plaster casts. Patient-reported outcomes supported this finding, with all studies qualitatively indicating improved perceived lightness, and no study reporting a preference for heavier conventional devices.

#### Cost outcomes

Cost outcomes showed the opposite trend, with conventional immobilization devices being more economical in the majority of comparative reports. Approximately 75% of comparative cost analyses (3 of 4 studies) demonstrated higher costs associated with 3D-printed devices, driven by material expenses and production workflow requirements.

#### Device production time

Production time followed a similar pattern, with 80% of comparative studies (4 of 5 studies) reporting longer production or workflow times for 3D systems. Average scan-to-device or fabrication times ranged from ~ 145 to 161 min (and up to ~ 2.85 h in full workflow analyses), compared with approximately 30–70 min for conventional cast application, representing roughly a 2–4 times longer production time for 3D systems, depending on workflow definition.

## Discussion

This systematic review and meta-analysis evaluated the clinical effectiveness, safety, and patient-reported outcomes of 3D-printed immobilization devices compared with conventional immobilization techniques for upper-extremity fractures across 12 studies and 445 participants. The findings suggest that 3D-printed immobilization is a clinically viable alternative to traditional immobilization methods, demonstrating comparative results in fracture healing, favorable patient satisfaction, and improved skin-related tolerability, while evidence for pain reduction and functional recovery remains clinically promising but statistically inconclusive.

3D-printed splints demonstrated the most clinically meaningful conclusions; however, this distribution reflects the available literature rather than a basis for direct comparison between intervention subgroups, highlighting the lack of true comparative studies addressing differences across 3D immobilization devices and varying population groups.

### Clinical significance

Across all outcome domains, the direction of effect consistently favored 3D-printed immobilization, and several findings carry meaningful clinical implications independent of formal statistical significance. Fracture healing success was reported at or near 100% across all studies where union was explicitly assessed, regardless of device type or population, suggesting that 3D-printed devices provide structurally adequate immobilization comparable to conventional plaster and fiberglass constructs. This is particularly relevant given that healing efficacy represents the foundational requirement for any immobilization device, and no study identified nonunion or loss of reduction attributable to 3D device failure.

Patient satisfaction represented the most consistently and robustly positive outcome domain, with 10 of 11 comparative and feasibility studies favoring 3D-printed immobilization across both adult and pediatric populations and across splint-, cast-, and orthosis-based designs. The improvements in satisfaction were attributed across studies to enhanced comfort, superior fit, improved hygiene access, reduced itching and odor, and lower cast-care burden, all of which represent clinically meaningful quality-of-life benefits during the immobilization period. Skin-related outcomes similarly demonstrated consistent and clinically relevant advantages for 3D-printed devices, with markedly lower rates of skin irritation, pressure complaints, and cast-care burden, particularly in pediatric populations, where Skibicki [21] demonstrated intact skin in 72.7% of 3D orthosis-treated limbs compared with only 16.7% of fiberglass cast-treated limbs.

Pain reduction and functional recovery demonstrated early advantages with 3D-printed immobilization that were clinically modest in absolute terms but directionally consistent. The early superiority in range of motion observed in the Ma trial (median ROM 30° vs 22°; *p* < 0.001) and the superior early DASH scores reported by Xiao [7, 8] suggest that the open-lattice and lighter-weight design of 3D devices may facilitate earlier mobilization and rehabilitation engagement. However, these early advantages were not sustained at longer follow-up intervals in higher-quality trials, including Guebeli [11], which found no significant differences in ROM, grip strength, DASH, or PRWE scores at 12, 26, or 52 weeks, suggesting that the long-term functional trajectory may converge regardless of immobilization method.

The future of immobilization may not depend solely on increasing rigidity or improving radiographic alignment, but rather on optimizing patient recovery and overall treatment experience. Recent evidence by Awad [29], comparing figure-of-eight bandages with arm slings in clavicle fractures, demonstrated that many differences between immobilization strategies, while statistically significant, may remain clinically modest. This persistent lack of consensus regarding optimal immobilization techniques supports a growing shift toward outcomes centered on comfort, usability, hygiene, adherence, and patient satisfaction [30]. Within this evolving paradigm, the consistent advantages demonstrated by 3D-printed immobilization devices across satisfaction and skin-related outcomes in the present review position them as a promising advancement in patient-centered fracture care, with a differentiated value proposition that extends beyond structural immobilization equivalence alone.

### Device properties outcomes (weight, cost, production time)

3D device properties, such as device weight, cost, and production time, were analyzed across studies and healthcare systems, showing heterogeneity that limits formal economic or cost-effectiveness conclusions.

Studies inconsistently reported material cost, unit cost, and workflow time across different currencies and production settings, without the inclusion of additional economic inputs such as labor, equipment depreciation, software costs, failed prints, or refitting requirements. While some studies suggested potential cost neutrality or modest savings, others reported higher direct costs for 3D printing, reflecting differences in scale, workflow design, and institutional context.

Production time was consistently longer for 3D-printed devices, largely due to scanning, design, and fabrication steps. However, variability in how time was defined (scan-to-print vs total workflow vs in-hospital production) limits comparability, and findings should be interpreted descriptively. One study, Factor [28], reported improved radiographic visualization with open-lattice designs, enabling fracture assessment without cast removal, although this remains descriptive and unquantified due to limited data.

Incorporating 3D printed immobilization technologies in clinical practice, which demonstrated lighter weight is associated with higher costs and longer production times in most comparative studies, reflecting workflow inefficiencies rather than differences in clinical effectiveness at this time.

However, it is worthy to note that these limitations appear to reflect early-stage workflow inefficiencies and variability in institutional adoption rather than differences in clinical effectiveness or downstream benefits. Reduced follow-up visits, improved imaging access, and fewer cast modifications were not captured and may partially offset initial production delays. While the technology shows clear ergonomic and patient-centered advantages, its feasibility for widespread clinical adoption will likely depend on workflow standardization, automation of design processes, and more comprehensive economic evaluations incorporating full cost accounting and downstream healthcare utilization.

### Statistical significance and meta-analytic findings

Despite the consistent clinical directionality of findings, formal statistical significance was not achieved in any of the three meta-analyses conducted. For pain outcomes, the pooled effect size was small-to-moderate (Hedges’ g = 0.36) but non-significant (95% CI [− 1.94, 2.66]; *p* = 0.295). For patient satisfaction, a large, pooled effect was identified (Hedges’ g = 0.83) that similarly did not reach significance (95% CI [− 0.21, 1.88]; *p* = 0.085). For functional outcomes, the pooled effect was negligible and non-significant (Hedges’ g = 0.13; 95% CI [− 1.02, 1.28]; *p* = 0.679). The failure to achieve statistical significance across meta-analyzed outcomes should be interpreted cautiously, as it reflects methodological and sample-size constraints and heterogeneous assessment tools rather than necessarily indicating the absence of a true treatment effect. The consistent directionality across independent studies, combined with statistically significant individual trial results, supports the interpretation that a clinically meaningful benefit exists but remains inadequately powered for definitive pooled confirmation.

### Risk of bias and evidence strength

RoB was assessed using Cochrane RoB 2 for randomized trials and ROBINS-I/JBI tools for nonrandomized studies and case series [31–34]. The risk of bias profile of the included evidence base has important implications for the certainty of conclusions that can be drawn from this review. The inability to blind participants or outcome assessors to immobilization device type was a common limitation across randomized trials and is of consequence for subjective outcomes such as pain and satisfaction, where expectation and experience effects may systematically inflate apparent treatment differences in favor of the novel intervention. The two randomized studies judged at high risk of bias, Khoury and Xu [23, 24], contributed to the meta-analyses for pain and satisfaction, respectively, and their inclusion may have introduced directional bias into the pooled estimates. Among non-randomized studies, the serious risk of bias judgment for Chen [26] limits any comparative inference from that study, and its data should be interpreted as hypothesis-generating only. The uniformly moderate risk of bias across feasibility studies similarly limits their contribution to effect estimation, as uncontrolled designs cannot adequately distinguish treatment effects from natural fracture recovery trajectories.

**Fig. 4:**
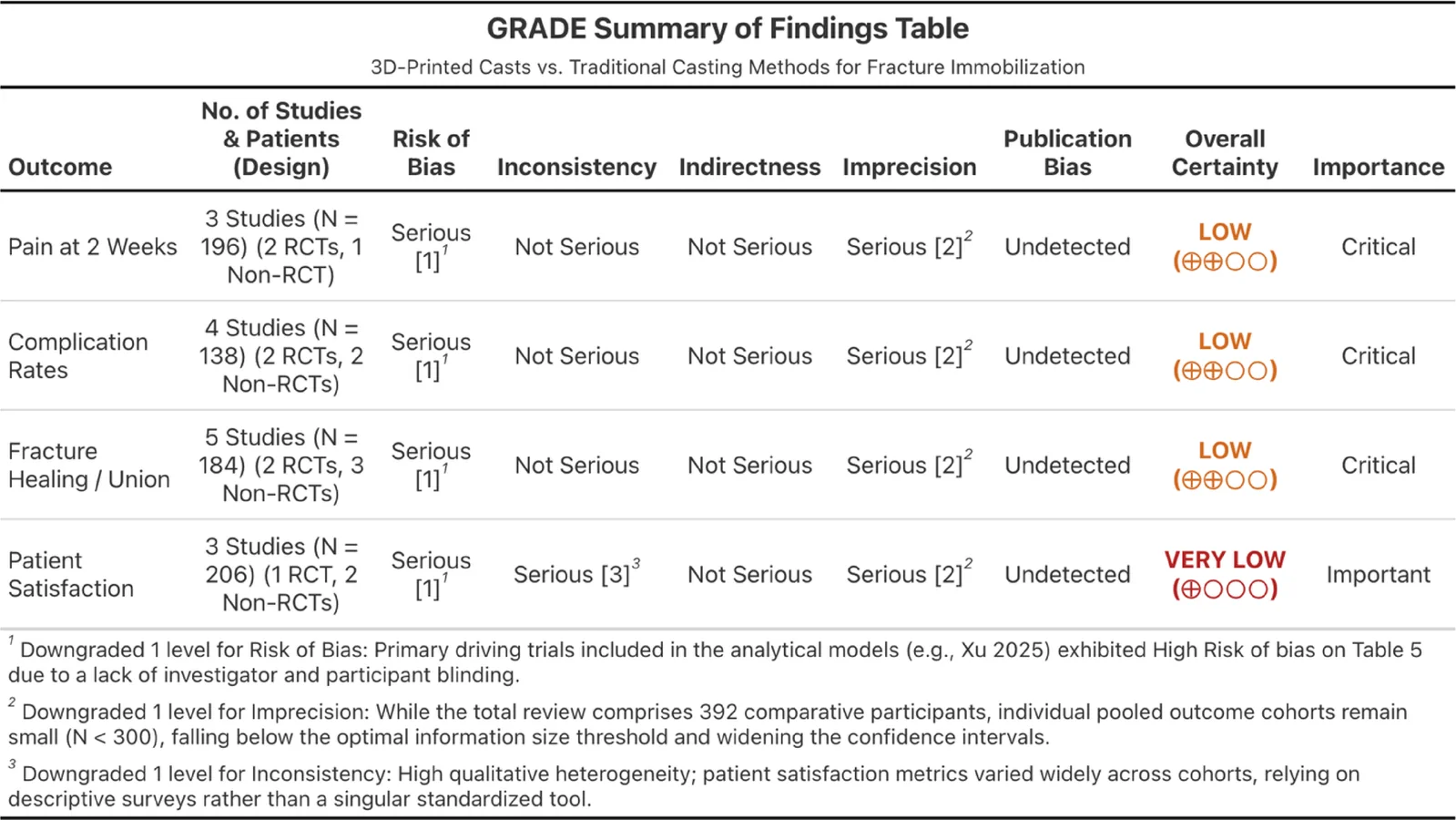
GRADE table

Applying a GRADE-informed framework, the certainty of evidence would be rated as low to very low for pain, functional, and complication outcomes, where pooled estimates were imprecise, and individual study bias was substantial (Fig. 4). Certainty for patient satisfaction and skin-related outcomes may be marginally higher, given the consistency of directional findings across study designs, populations, and device types, but would not exceed low-to-moderate in the absence of low-risk-of-bias trials and given the reliance on heterogeneous and non-validated measurement instruments. These considerations indicate that while the directional findings of this review are informative and hypothesis-generating, they should not be interpreted as definitive evidence of superiority, and clinical adoption decisions should be made with explicit recognition of the current evidence uncertainty. Adequately powered randomized trials conducted at low risk of bias, with standardized outcome measurement and extended follow-up, are necessary before firm conclusions can be drawn regarding the comparative effectiveness of 3D-printed immobilization devices.

## Limitations

The heterogeneity of outcome measurement instruments across studies limited comprehensive quantitative pooling and necessitated reliance on narrative synthesis for several outcome domains. Publication bias cannot be excluded given the small number of studies and the general tendency for positive findings to be more readily published, particularly in the context of novel medical devices. The predominance of adult populations and limited studies on the pediatric population limit the strength of conclusions applicable to pediatric patients, who represent an important clinical subgroup given the higher relative frequency of forearm and wrist fractures in children [35]. Finally, the variability in 3D-printed device designs, materials, and fabrication workflows across studies introduces a form of intervention heterogeneity that limits the ability to attribute outcomes to any specific device characteristic, such as lattice geometry, material rigidity, or fit precision [14, 36].

### Heterogeneity

Despite systematic efforts to homogenize findings through a structured narrative synthesis stratified by population and device type, heterogeneity remained pervasive across all outcome domains and could not be fully resolved by stratification alone.

While this approach identified broadly consistent directional trends favoring 3D-printed immobilization, the limited meta-analytic pooling that was feasible still demonstrated substantial between-study heterogeneity for patient satisfaction (I^2^ = 77.50%; *p* = 0.005), moderate heterogeneity for functional outcomes (I^2^ = 51.65%; *p* = 0.128), and absent heterogeneity for pain (I^2^ = 0.00%; *p* = 0.380). The high satisfaction heterogeneity reflects the use of disparate, largely non-validated measurement scales across adult and pediatric cohorts and across device types, each of which may influence patient experience through different mechanisms.

Beyond statistical heterogeneity, methodological heterogeneity in assessment tools across all domains encompassing different pain scales, functional indices, satisfaction instruments, and skin assessment methods further limited eligible study arms for pooling to between two and four per outcome. Adoption of standardized outcome instruments and pre-specified subgroup analyses in future trials will be essential to enable more robust pooling and reduce interstudy variability.

### Small sample size and statistical power

A critical limitation of the current evidence base is the consistently small sample sizes across included studies, with a median sample size of approximately 32 participants and half of all studies enrolling 25 or fewer participants. This severely constrains the statistical power of individual studies and the precision of pooled estimates, as evidenced by the wide confidence intervals spanning the null in all three meta-analyses. The Knapp–Hartung adjustment applied in all meta-analyses, while appropriate for small numbers of studies and protective against type I error, further contributed to conservative confidence interval estimation, particularly in the satisfaction meta-analysis, where substantial heterogeneity compounded imprecision. The narrow pooling of only two to four study arms per meta-analysis additionally limits the generalizability of pooled estimates. Adequately powered multicenter randomized trials with pre-specified sample size calculations based on clinically meaningful effect sizes are necessary to generate definitive pooled evidence for or against 3D-printed immobilization across individual outcome domains.

### Short follow-up duration

The duration of follow-up was limited across the majority of included studies, with most reporting outcomes at 2 to 12 weeks and few extending beyond 6 weeks of post-immobilization assessment. Only Guebeli [11] provided follow-up to 52 weeks, representing the sole study with sufficient duration to evaluate long-term functional equivalence, ROM, and patient-reported outcomes. The predominance of short follow-up intervals means that the current evidence base is well-suited to characterizing the early immobilization experience, including pain, skin tolerance, satisfaction, and early functional recovery. However, it is insufficient to conclude long-term fracture remodeling, late functional outcomes, or late device-related complications. This is particularly relevant for pediatric populations, where growth-related remodeling and long-term skeletal outcomes require extended surveillance beyond the immobilization period.

## Conclusions

The current evidence suggests that 3D-printed immobilization devices in upper extremity fractures show comparable represent a clinically viable and patient-preferred alternative to conventional casting and splinting for upper-extremity fractures, demonstrating comparable fracture healing, superior patient satisfaction and skin tolerability, and early functional advantages that may not persist to long-term follow-up. However, the evidence base is characterized by small sample sizes, short follow-up durations, moderate-to-high risk of bias, and substantial interstudy heterogeneity, collectively limiting the certainty of pooled estimates and limited definitive clinical recommendations at this time. Adequately powered, long-term randomized trials stratified by device type and patient population are needed to establish the role of 3D-printed immobilization within evidence-based fracture management protocols.

## Data Availability

All data generated or analyzed during this study are included in this published article and its supplementary information files. The extracted data supporting the findings of this study are available from the corresponding author on reasonable request.

## References

[1] Karl JW, Olson PR, Rosenwasser MP. The epidemiology of upper extremity fractures in the United States, 2009. J Orthop Trauma. 2015;29(8):e242-244. 10.1097/BOT.0000000000000312. 25714441

[2] Ottenhoff J, Kongkatong M, Hewitt M, Phillips J, Thom C. A narrative review of the uses of ultrasound in the evaluation, analgesia, and treatment of distal forearm fractures. J Emerg Med. 2022;63(6):755–65. 10.1016/j.jemermed.2022.09.012. 36351851

[3] de Putter CE, Selles RW, Haagsma JA, Polinder S, Panneman MJM, Hovius SER, et al. Health-related quality of life after upper extremity injuries and predictors for suboptimal outcome. Injury. 2014;45(11):1752–8. 10.1016/j.injury.2014.07.016. 25150751

[4] Ekanayake C, Gamage JCPH, Mendis P, Weerasinghe P. Revolution in orthopedic immobilization materials: a comprehensive review. Heliyon. 2023;9(3):e13640. 10.1016/j.heliyon.2023.e13640. 36915506 PMC10006541

[5] Scheinberg M, Nihalani S, Mehta L, Shah A. Evolution in casting techniques: a narrative review of casting methods, techniques, and innovation. Cureus. 2024. 10.7759/cureus.53454. 38435235 PMC10908428

[6] Yan ET, Chung D. Casting a new light: a systematic review of 3D-printed casting for non-surgical management of orthopedic injuries. J Orthop Exp Innov. 2025. 10.60118/001c.143914.

[7] Ma H, Ruan B, Li J, Zhang J, Wu C, Tian H, et al. Topology-optimized splints versus casts for distal radius fractures: a randomized clinical trial. JAMA Netw Open. 2024;7(2):e2354359. 10.1001/jamanetworkopen.2023.54359. 38306099 PMC10837751

[8] Xiao YP, Xu HJ, Liao W, Li ZH. Clinical application of instant 3D printed cast versus polymer orthosis in the treatment of colles fracture: a randomized controlled trial. BMC Musculoskelet Disord. 2024;25(1):104. 10.1186/s12891-024-07212-8. 38297262 PMC10829219

[9] Tobler-Ammann B, Schuind F, Voillat L, Gentilhomme T, Vögelin E, Murith N, et al. Developing 3D-printed wrist splints for distal radius and scaphoid fractures. J Wrist Surg. 2024;13(05):390–7. 10.1055/s-0044-1779053. 39296652 PMC11407844

[10] Atallah H, Qufabz T, Bakhsh HR, Ferriero G. The current state of 3D-printed orthoses clinical outcomes: a systematic review. BMC Musculoskelet Disord. 2025;26(1):822. 10.1186/s12891-025-09070-4. 40890671 PMC12400727

[11] Guebeli A, Thieringer F, Honigmann P, Keller M. In-house 3D-printed custom splints for non-operative treatment of distal radial fractures: a randomized controlled trial. J Hand Surg Eur Vol. 2024;49(3):350–8. 10.1177/17531934231187554. 37458129

[12] Keller M, Guebeli A, Thieringer F, Honigmann P. Overview of in-hospital 3D printing and practical applications in hand surgery. BioMed Res Int. 2021;2021(1):4650245. 10.1155/2021/4650245. 33855068 PMC8019389

[13] Lazzeri S, Talanti E, Basciano S, Barbato R, Fontanelli F, Uccheddu F, et al. 3D-printed patient-specific casts for the distal radius in children: outcome and pre-market survey. Materials. 2022;15(8):2863. 10.3390/ma15082863. 35454555 PMC9027121

[14] Fong FJY, Onggo JD, Munro YL, Yam MGJ. Applications of 3D printing in orthopedics: a scoping review. Eur J Orthop Surg Traumatol. 2025;35(1):367. 10.1007/s00590-025-04429-8. 40884598

[15] Hellman S, Frisch P, Platzman A, Booth P. 3D Printing in a hospital: Centralized clinical implementation and applications for comprehensive care. Digit Health. 2023;9:20552076231221900. 10.1177/20552076231221899. 38130801 PMC10734340

[16] Schwartz DA, Schofield KA. Utilization of 3D printed orthoses for musculoskeletal conditions of the upper extremity: a systematic review. J Hand Ther. 2023;36(1):166–78. 10.1016/j.jht.2021.10.005. 34819255

[17] Higgins MJ, Gomez RW, Storino M, Wang N, Strouse A, Zook Z, et al. 3D-printed casts improve patient-reported function and satisfaction in children with stable fracture patterns. J Pediatr Orthop. 2025;45(9):546–51. 10.1097/BPO.0000000000003009. 40700461

[18] Ganapathy A, Chen D, Elumalai A, Albers B, Tappa K, Jammalamadaka U, et al. Guide for starting or optimizing a 3D printing clinical service. Methods. 2022;206:41–52. 10.1016/j.ymeth.2022.08.003. 35964862

[19] Lu P, Liao Z, Zeng Q, Chen H, Huang W, Liu Z, et al. Customized three-dimensional-printed orthopedic close contact casts for the treatment of stable ankle fractures: finite element analysis and a pilot study. ACS Omega. 2021;6(4):3418–26. 10.1021/acsomega.0c06031. 33553960 PMC7860236

[20] Schlégl Á, Told R, Kardos K, Szőke A, Ujfalusi Z, Maróti P. Evaluation and comparison of traditional plaster and fiberglass casts with 3D-printed PLA and PLA–CaCO3 composite splints for bone-fracture management. Polymers. 2022;14(17):3571. 10.3390/polym14173571. 36080645 PMC9460134

[21] Skibicki HE, Katt BM, Lutsky K, Wang ML, McEntee R, Vaccaro AR, et al. Three dimensionally printed versus conventional casts in pediatric wrist fractures. Cureus. 2021. 10.7759/cureus.19090. 34868748 PMC8626708

[22] Tobler-Ammann B, Schuind F, Voillat L, Vögelin E. Acceptability and safety of 3D printed wrist-based orthoses compared to fiberglass casts for the treatment of non-surgical distal radius- and scaphoid fractures: a randomized feasibility trial. J Hand Ther. 2025;38(1):143–51. 10.1016/j.jht.2024.11.004. 39755483

[23] El Khoury G, Libouton X, De Boeck F, Barbier O. Use of a 3D-printed splint for the treatment of distal radius fractures: a randomized controlled trial. Orthop Traumatol Surg Res. 2022;108(5):103326. 10.1016/j.otsr.2022.103326. 35595196

[24] Xu C, Wang L, Zhang M, Li X, Li K. A clinical study on the application of three-dimensionally printed splints combined with finite element analysis in paediatric distal radius fractures. Front Pediatr. 2025;13:1559762. 10.3389/fped.2025.1559762. 40248025 PMC12003414

[25] Chen Y, Lin H, Yu Q, Zhang X, Wang D, Shi L, et al. Application of 3D-printed orthopedic cast for the treatment of forearm fractures: finite element analysis and comparative clinical assessment. BioMed Res Int. 2020;2020(1):9569530. 10.1155/2020/9569530. 32775455 PMC7399740

[26] Chen YJ, Lin H, Zhang X, Huang W, Shi L, Wang D. Application of 3D–printed and patient-specific cast for the treatment of distal radius fractures: initial experience. 3D Print Med. 2017;3(1):11. 10.1186/s41205-017-0019-y. 29782603 PMC5954789

[27] Surucu S, Aydın M, Güray Batma A, Karaşahin D, Mahiroğulları M. Evaluation of the patient satisfaction of using a 3D printed medical casting in fracture treatment. Jt Dis Relat Surg. 2022;33(1):180–6. 10.52312/jdrs.2022.372. 35361093 PMC9057558

[28] Factor S, Atlan F, Pritsch T, Rumack N, Golden E, Dadia S. In-hospital production of 3D-printed casts for non-displaced wrist and hand fractures. SICOT-J. 2022;8:20. 10.1051/sicotj/2022021. 35608413 PMC9128606

[29] Awad G, Boutros M, Asmar C, Hanna T. Figure-of-eight bandage versus arm sling for the conservative treatment of midshaft clavicle fractures in adults: a meta-analysis. Indian J Orthop. 2026;60(2):311–24. 10.1007/s43465-025-01593-1PubMedPMID:41669212;PubMedCentralPMCID:PMC12883619. 41669212 PMC12883619

[30] Awad G, Boutros M, Asmar C, Hanna T. Functional bracing versus rigid plaster casting for the immobilization of colles fractures in adults: a meta-analysis of randomized controlled trials. Hand. 2025;30:15589447251389660. 10.1177/15589447251389659PubMedPMID:41320967;PubMedCentralPMCID:PMC12669006. PMC12669006 41320967

[31] Higgins J, Thomas J, Chandler J, Cumpston M, Li T, Page M. Cochrane handbook for systematic reviews of interventions. Cochrane; 2024. 10.1002/14651858.ED000142 PMC10284251 31643080

[32] Munn Z, Barker TH, Moola S, Tufanaru C, Stern C, McArthur A, et al. Methodological quality of case series studies: an introduction to the JBI critical appraisal tool. JBI Evid Synth. 2020;18(10):2127–33. 10.11124/JBISRIR-D-19-00099. 33038125

[33] Sterne JA, Hernán MA, Reeves BC, Savović J, Berkman ND, Viswanathan M, et al. ROBINS-I: a tool for assessing risk of bias in non-randomised studies of interventions. BMJ. 2016. 10.1136/bmj.i4919. 27733354 PMC5062054

[34] Sterne JAC, Savović J, Page MJ, Elbers RG, Blencowe NS, Boutron I, et al. RoB 2: a revised tool for assessing risk of bias in randomised trials. BMJ. 2019. 10.1136/bmj.l4898. 31462531

[35] Nellans KW, Kowalski E, Chung KC. The epidemiology of distal radius fractures. Hand Clin. 2012;28(2):113–25. 10.1016/j.hcl.2012.02.001PubMedPMID:22554654;PubMedCentralPMCID:PMC3345129. 22554654 PMC3345129

[36] Prządka M, Pająk W, Kleinrok J, Pec J, Michno K, Karpiński R, et al. Advances in 3D printing applications for personalized orthopedic surgery: from anatomical modeling to patient-specific implants. J Clin Med. 2025;14(11):3989. 10.3390/jcm14113989. 40507750 PMC12156138

